# A Case of Synchronous and Metachronous Gastric Neoplasms Associated With Autoimmune Gastritis

**DOI:** 10.7759/cureus.79929

**Published:** 2025-03-02

**Authors:** Kimitoshi Kubo, Issei Ashida, Noriko Kimura

**Affiliations:** 1 Department of Gastroenterology, National Hospital Organization Hakodate Medical Center, Hakodate, JPN; 2 Department of Gastroenterology, Hakodate Medical Center, Hakodate, JPN; 3 Department of Diagnostic Pathology, National Hospital Organization Hakodate Medical Center, Hakodate, JPN

**Keywords:** autoimmune gastritis, clinical pathology, endoscopic treatment, extensive mucosal atrophy, gastric adenoma, gastric cancer, metachronous cancers, risk factors, synchronous cancers

## Abstract

Patients with autoimmune gastritis (AIG) are reported to be associated with an increased risk of developing gastric neuroendocrine and gastric tumors. Again, those with cancer are shown to be at risk of developing multiple primary cancers within two months of the first primary cancer (synchronous cancers) or more than two months afterward (metachronous cancers). A 78-year-old man was diagnosed with early gastric cancer and referred to our hospital for endoscopic treatment. Curative resection was performed with endoscopic submucosal dissection (ESD), which also revealed AIG in the background gastric mucosa. Follow-up esophagogastroduodenoscopy (EGD) performed three months later revealed an erythematous, superficial depressed lesion and a whitish, superficial flat lesion in the greater curvature of the gastric angle, which established the diagnosis of early gastric cancers. Curative resection was again performed with ESD. Retrospectively, one of these lesions was found to be a synchronous gastric cancer. A follow-up EGD performed one year later newly detected a 5 mm adenoma in the gastric angle, which was treated endoscopically as a metachronous lesion. Thus, the present case highlights the need to watch for multiple primary cancers when treating patients with cancer, particularly those with AIG.

## Introduction

Autoimmune gastritis (AIG) is a subtype of gastritis involving the destruction of parietal cells by autoimmune mechanisms, resulting in the production of anti-parietal cell antibodies and/or anti-intrinsic factor antibodies [1]. AIG not only is recognized as a rare condition with a reported prevalence of 0.3%-2.7% [2] but remains asymptomatic until the late stage in most patients [3]. The associated atrophy of the oxyntic mucosa can lead to the malabsorption of iron and vitamin B12, thus accounting for anemia and neuropathy [4]. Additionally, AIG reportedly represents a preneoplastic condition potentially resulting in type I neuroendocrine tumors and gastric cancers [4].

While *Helicobacter pylori* infection is shown to be predominantly associated with the risk of gastric adenocarcinoma and to account for >90% of all gastric cancers, AIG is also shown to be a non-negligible risk factor responsible for chronic gastric inflammation, leading to gastric atrophy and metaplasia [5]. Of note, gastric atrophy represents a key step in the development of gastric neoplasms and is also reported to be associated with intestinal gastric cancer [6]. Clinicopathologically, early gastric cancers associated with AIG have been characterized by Kitamura et al. [7] as affecting a high proportion of females as protruded-type, large, papillary tumors occurring in upper locations and by Nomura et al. [8] as accounting for a high proportion of multiple gastric cancers (synchronous and metachronous (38.0%)) and occurring as protruded-type lesions in upper regions and the greater curvature.

We herein report a case of synchronous and metachronous gastric neoplasms associated with AIG, which was amenable to complete resection with endoscopic submucosal dissection (ESD).

## Case presentation

**Figure 1:**
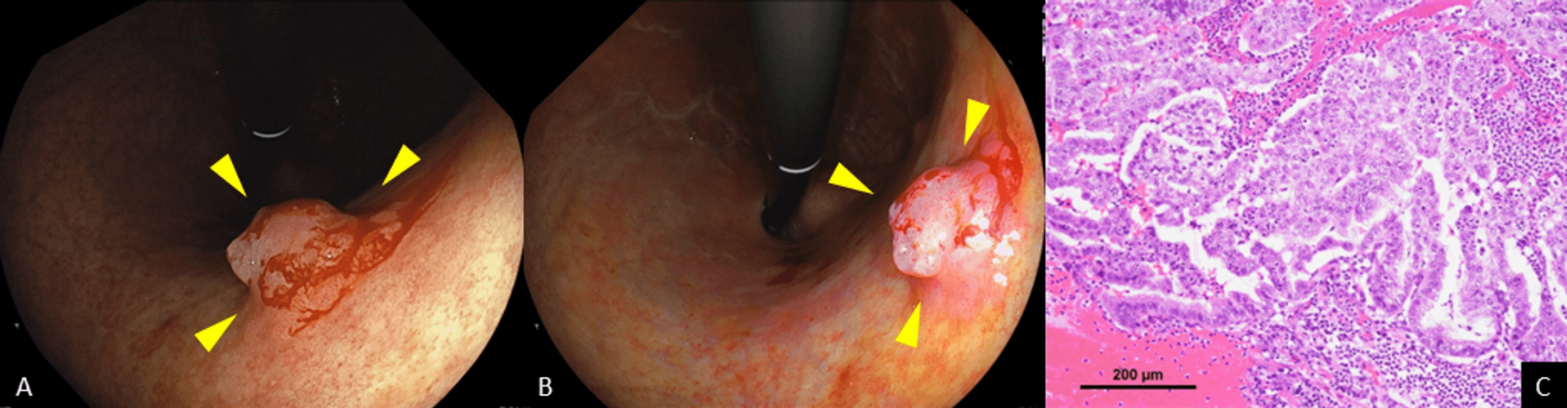
Endoscopic and histopathological findings A: WLI depicted a 15 mm elevated lesion in the posterior wall of the gastric middle body (arrowheads). B: Linked color imaging depicted a purple-colored, elevated lesion with its central area shown to be orangish-colored (arrowheads). C: Histological examination showed the lesion to be an adenocarcinoma, papillary, Paris type 0-I, measuring 14 × 13 mm, pT1a (M), with no lymphovascular invasion. WLI: white light imaging

A 78-year-old asymptomatic man underwent a screening esophagogastroduodenoscopy (EGD) at a nearby hospital. He had a history of cerebral infarction, hypertension, and atrial fibrillation but no history of *H. pylori* eradication. EGD incidentally revealed a 15 mm elevated lesion in the posterior wall of the gastric middle body on white light imaging (WLI) (Figure 1A). The lesion was depicted as a purple-colored, elevated lesion with its central area shown to be orangish-colored using linked color imaging (Figure 1B), which biopsy revealed as an adenocarcinoma. He was diagnosed with early gastric cancer and referred to our hospital for endoscopic treatment. Laboratory data showed the patient to be positive for anti-parietal cells but negative for serum *H. pylori* IgG antibody, thus suggesting the presence of AIG. Endoscopic submucosal dissection (ESD) was performed, and a histological examination of the ESD specimens showed the lesion to be an adenocarcinoma, papillary, Paris type 0-I, measuring 14 × 13 mm, pT1a (M), with no lymphovascular invasion (Figure 1C).

**Figure 2:**
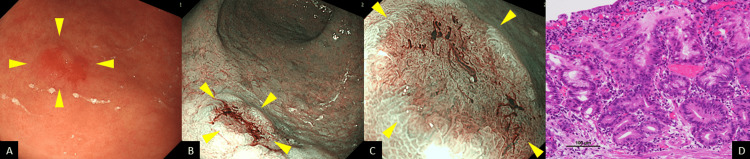
Follow-up EGD and histopathological findings A: Follow-up EGD performed three months later revealed an erythematous, superficial, depressed lesion (arrowheads). B and C: The two lesions were depicted as well-circumscribed, superficial lesions on NBI with an irregular microvascular pattern shown to be present within the demarcation line on magnifying NBI (arrowheads). D: Histological examination revealed one lesion to be a moderately differentiated adenocarcinoma, Paris type 0-IIc, measuring 8 × 4 mm, pT1a (M), with no lymphovascular invasion. EGD: esophagogastroduodenoscopy, NBI: narrow-band imaging

**Figure 3:**
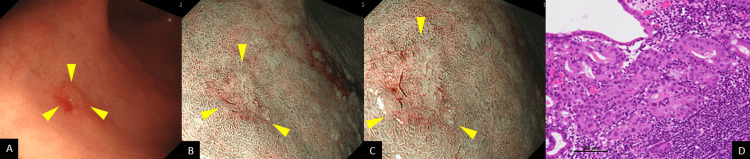
Follow-up EGD and histopathological findings A: Follow-up EGD performed three months later also revealed another whitish, superficial flat lesion in the greater curvature of the gastric angle on WLI (arrowheads). B and C: The two lesions were also depicted as well-circumscribed superficial lesions on NBI each with an irregular microvascular pattern shown to be present within the demarcation line on magnifying NBI (arrowheads). D: Histological examination revealed a moderately differentiated adenocarcinoma, Paris type 0-IIb, measuring 2 × 2 mm, pT1a (M), with no lymphovascular invasion. EGD: esophagogastroduodenoscopy, WLI: white light imaging, NBI: narrow-band imaging

**Figure 4:**
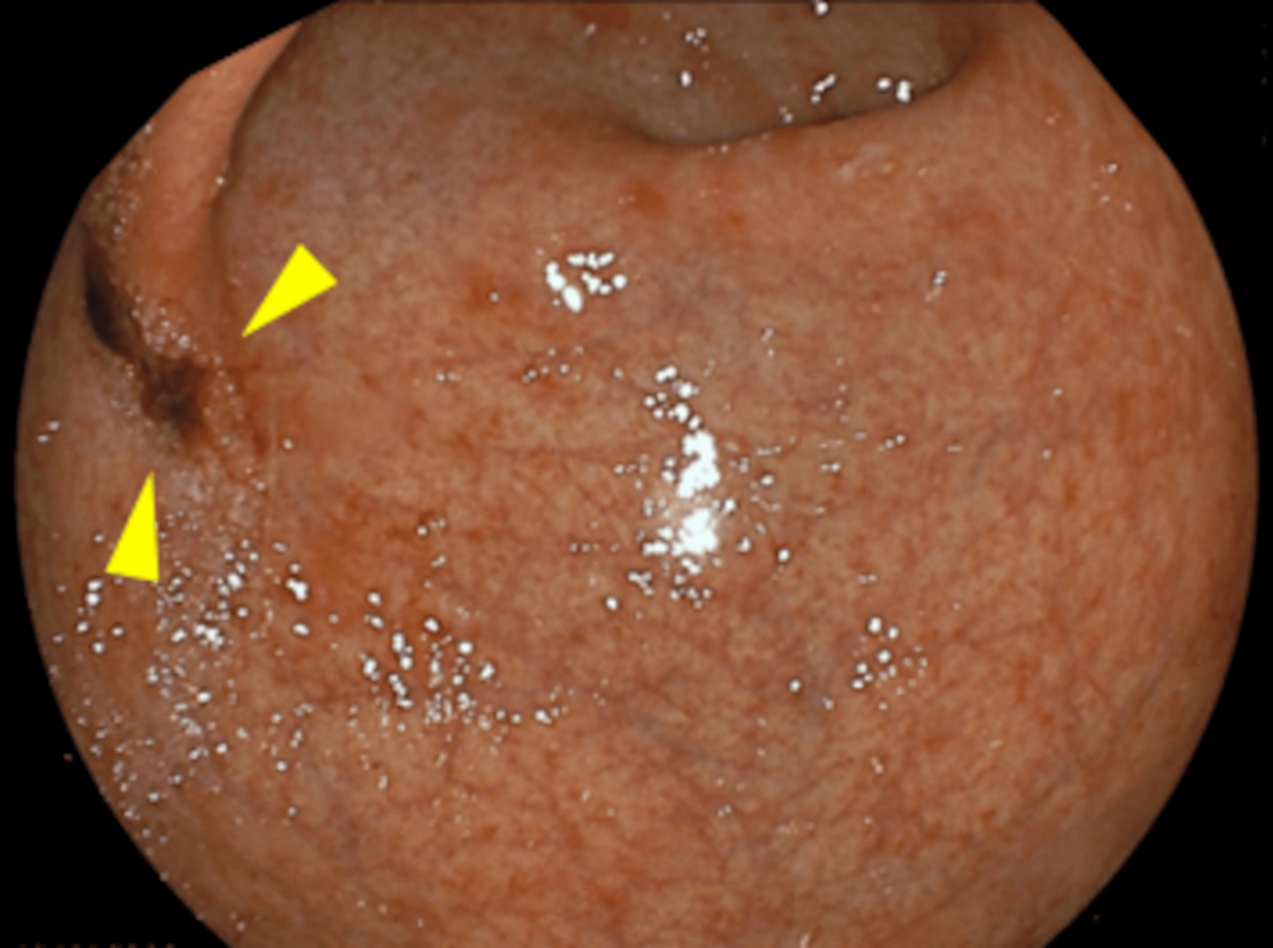
Retrospective finding It was found, retrospectively, that a 0-IIc lesion had been inadvertently missed by the initial EGD (arrowheads). EGD: esophagogastroduodenoscopy

Follow-up EGD performed three months later revealed an erythematous, superficial depressed lesion (Figure 2A) and a whitish, superficial flat lesion (Figure 3A) in the greater curvature of the gastric angle on WLI. Again, the two lesions were depicted as well-circumscribed superficial lesions on narrow-band imaging (NBI) (Figure 2B and Figure 3B) with an irregular microvascular pattern shown to be present within the demarcation line on magnifying NBI (Figure 2C and Figure 3C). Based on these findings, each of these lesions was deemed consistent with the diagnosis of early gastric cancer. An EGD biopsy revealed adenocarcinoma in both lesions. The background gastric mucosa was deemed associated with atrophic gastritis O4 (O-P) based on the modified Kimura-Takemoto classification [9]. In addition, an examination of the biopsy specimens revealed proximal-predominant gastric mucosal atrophy with no evidence of *H. pylori* colonization. ESD was performed, and a histological examination of the ESD specimens revealed the two lesions to be as follows: a moderately differentiated adenocarcinoma, Paris type 0-IIc, measuring 8 × 4 mm, pT1a (M), with no lymphovascular invasion (Figure 2D) and a moderately differentiated adenocarcinoma, Paris type 0-IIb, measuring 2 × 2 mm, pT1a (M), with no lymphovascular invasion (Figure 3D). It was found, retrospectively, that a 0-IIc lesion had been inadvertently missed by the initial EGD (Figure 4).

**Figure 5:**
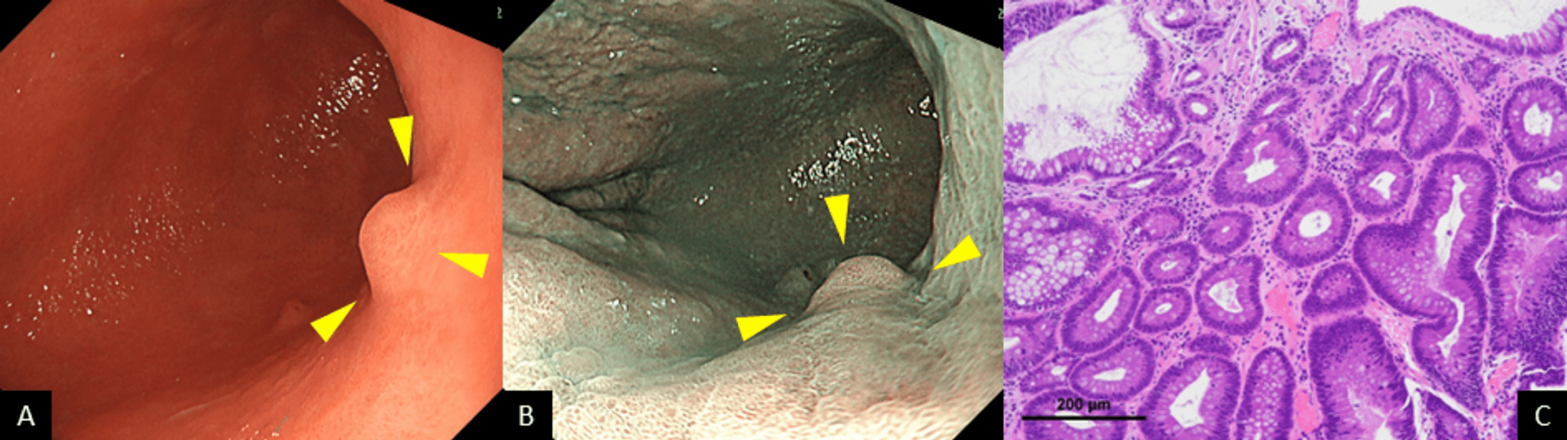
Follow-up EGD and histopathological findings A and B: Follow-up EGD performed one year later newly revealed a 5 mm whitish, elevated lesion in the posterior wall of the gastric angle on WLI and NBI (arrowheads). C: Histological examination confirmed the lesion to be an adenoma. EGD: esophagogastroduodenoscopy, WLI: white light imaging, NBI: narrow-band imaging

Follow-up EGD performed one year later newly revealed a 5 mm, whitish, elevated lesion in the posterior wall of the gastric angle on WLI and NBI (Figure 5A, 5B), which biopsy revealed as an adenoma. Endoscopic mucosal resection was performed, and a histological examination of the resected specimen confirmed the lesion to be an adenoma (Figure 5C).

The patient was thus diagnosed with synchronous and metachronous neoplasms associated with AIG. After discharge, the patient has been visiting our hospital regularly for three years, and follow-up EGD and computed tomography examinations have shown no evidence of recurrence to date.

## Discussion

In patients with gastric cancer associated with AIG, clinicians should be aware of their risk of developing synchronous or metachronous gastric neoplasms, given that patients with AIG are reported to have three times higher risk of developing gastric cancer than the general population [10,11]. Indeed, the European Management of Precancerous Conditions and Lesions in the Stomach (MAPS) guidelines [12] recommend three-year endoscopic follow-up for all patients presenting with extensive atrophy (stages III and IV of the Operative Link for Gastritis Assessment (OLGA) classification [13]). Similarly, the American Gastroenterological Association (AGA) clinical practice update recommends three-year endoscopic follow-up for patients presenting with advanced atrophic gastritis [11]. The reported risk factors for gastric cancer include pernicious anemia, extensive atrophy, long-standing AIG, intestinal metaplasia, and age ≥ 50 years [8,14]. A multicenter study of AIG patients with severe atrophy, positive antibody or pernicious anemia, and hypergastrinemia reported a higher prevalence of gastric cancer in these patients than previously reported at 9.8% (24/245) [15]. Another study of AIG patients with metachronous gastric neoplasms described their characteristics as having a mean age of 65.1 years, being mainly males (accounting for 71%), and presenting with atrophic gastritis (O-P) [16]. Again, AIG patients are shown to have a significantly higher incidence of metachronous gastric neoplasms following endoscopic resection than non-AIG patients (45.0% versus 18.3%), suggesting the need to closely monitor and appropriately manage patients with AIG to facilitate early detection and management of recurrent lesions [16]. Therefore, it was speculated that advanced age and extensive atrophy, characteristic of late-stage AIG, would constitute risk factors for the development of AIG-associated synchronous and metachronous gastric neoplasms.

In the present case, we first treated the patient for the early gastric cancer as requested. It was deemed important, however, that attention be given to the background gastric mucosa that could promote carcinogenesis. Indeed, laboratory data led to AIG being suspected, and thus, detailed endoscopic observation, including biopsy, was performed during follow-up, which led to the diagnosis of AIG. In addition, the attending physician's awareness of the risks and clinicopathological features of AIG led to early detection and treatment of the synchronous and metachronous gastric neoplasms as well as the metachronous adenoma involved, which was deemed a precancerous lesion, based on the current guidelines [17].

## Conclusions

The present case demonstrates that synchronous and metachronous gastric neoplasms may be found to occur in patients with AIG. Thus, clinicians are well advised to watch for these lesions when treating patients with gastric neoplasms associated with AIG who are at risk, i.e., being at an advanced age or associated with extensive atrophy.
